# SLAMF8 and NINJ2 promote neuroinflammation and oxidative stress through TLR4 NF kappa B pathway in Alzheimer’s disease

**DOI:** 10.1038/s41598-025-02097-6

**Published:** 2025-05-20

**Authors:** Shuo Liu, Yuze He, He Chen, Wenwen Zhao, Heman Xu, Tao Bai, Juan Feng

**Affiliations:** 1 The Fourth People’s Hospital of Shenyang, Shenyang, Liaoning China; 2 Shengjing Hospital of China Medical University, China Medical University, Shenyang, China

**Keywords:** Alzheimer’s disease (AD), SLAMF8, NINJ2, TLR4/NF-κB pathway, Neuroinflammation, Oxidative stress, Therapeutic targets, Cell biology, Neuroscience, Medical research

## Abstract

This study aimed to investigate the expression and role of Signaling Lymphocytic Activation Molecule Family Member 8 (SLAMF8) in Alzheimer’s disease (AD), particularly its interaction with NINJ2 in the TLR4/NF-κB signaling pathway. SLAMF8 expression levels in AD models were analyzed using the Gene Expression Omnibus (GEO) database. Validation of SLAMF8 expression was conducted through RT-PCR and Western blot in Aβ_1−42_-exposed SH-SY5Y cells, LPS-exposed HMC3 cells, and APP/PS1 transgenic mice. The interaction between SLAMF8 and NINJ2 was explored through co-immunoprecipitation, confocal immunofluorescence, and Western blot analyses, focusing on their roles in the TLR4/NF-κB signaling pathway. SLAMF8 was significantly upregulated in AD models, and its overexpression activated the TLR4/NF-κB signaling pathway, leading to increased levels of pro-inflammatory cytokines and oxidative stress. NINJ2 was identified as a direct functional partner of SLAMF8, with co-localization observed in the cytoplasm. Knockout of NINJ2 abolished SLAMF8-mediated activation of the TLR4/NF-κB pathway, neuroinflammation, and oxidative stress. The SLAMF8-NINJ2-TLR4/NF-κB axis is a crucial signaling pathway in AD progression. SLAMF8 and NINJ2 emerge as potential therapeutic targets for managing AD, with significant implications for future research and clinical interventions.

**Supplementary Information:**

The online version contains supplementary material available at 10.1038/s41598-025-02097-6.

## Introduction

Alzheimer’s Disease (AD) is a major global health disorder, ranking among the most prevalent neurodegenerative disorders and a primary contributor to dementia-related mortality on a global scale^1^. AD is a complex and highly heterogeneous disease, arising from multiple interactions among genetic alterations, epigenetic changes, and environmental factors. Despite extensive research, the exact pathogenesis of AD remains elusive. However, there is substantial evidence suggesting that neuroinflammation and oxidative stress are closely associated with AD^2–4^. Furthermore, studies have indicated that AD patients might benefit from treatments targeting inflammation^2^ and oxidative stress^5^.

Members of the Signaling Lymphocytic Activation Molecule Family 8 (SLAMF8) have been recognized as crucial contributors to immune responses and inflammation. By modulating immune cell activity via pathways such as TLR4/NF-κB, PI3K/AKT, and MAPK, SLAMF8 influences the production of pro-inflammatory cytokines, including IL-6 and TNF-α^6–8^. Furthermore, SLAMF8 has been shown to amplify immune responses by regulating these cytokines^9,10^. These cytokines are known to play a critical role in driving neuroinflammation, a hallmark of Alzheimer’s Disease (AD). Thus, SLAMF8 may serve as a pivotal modulator in the inflammatory processes underlying AD pathology. However, despite these indications, the precise role of SLAMF8 in the progression of AD remains insufficiently characterized.

Nerve Injury-Treated Protein (NINJ2) is an adhesion molecule involved in neuron growth and regeneration through homophilic interactions^11^. There is evidence linking it to many neurological disorders such as stroke^12,13^, multiple sclerosis^14^, and Alzheimer’s disease^15^. Within the framework of inflammation, NINJ2 has been recognized as a mediator that promotes inflammation, as well as contributing to the activation of endothelial cells through the TLR4/NF-κB pathway^16^.

This study confirmed the elevated expression of SLAMF8 in Alzheimer’s disease (AD) by the analysis of data from the Gene Expression Omnibus (GEO) database. Additionally, we conducted a comprehensive evaluation of the expression levels of SLAMF8 in both Alzheimer’s disease (AD) cell and animal models. Furthermore, we examined the impact of SLAMF8 regulation on neuroinflammation and oxidative stress. In addition, we examined the relationship between SLAMF8 and NINJ2, investigating the impact of this interaction on the TLR4/NF-κB signaling complex. These results indicate that SLAMF8 may be a promising therapeutic target for Alzheimer’s disease.

## Methods

### Collection and preprocessing of data

The mRNA expression patterns of human Alzheimer’s disease (AD) were obtained from the Gene Expression Omnibus (GEO) database. In this study, we utilized GSE122063 to build co-expression networks and pinpoint hub genes associated with Alzheimer’s disease (AD). The microarray dataset included gene expression profiles captured in percutaneous allograft biopsies from 12 patients with Alzheimer’s disease (AD) and 11 controls without dementia. Normalization of the data was performed using the Robust Multiarray Average (RMA) technique, followed by log2 transformation and quantile normalization.

### Differentially expressed genes screening and co-expression network construction

After applying background correction and normalization, differentially expressed genes (DEGs) were detected using the “limma” R package (v3.56.2). The co-expression network was built using the “WGCNA” R package (v1.72-5), with a threshold of 0.85 for the correlation coefficient and a soft-thresholding power equivalent to 12. A cut height of 0.2 was established to combine modules with comparable characteristics, and the pink module was shown to be most strongly linked to AD.

### Functional enrichment and hub genes identification

The elevated genes inside the pink module were subjected to Gene Ontology (GO) enrichment analysis using the “enrichR” package. The obtained findings were then shown using the “ggplot2” R package. The Cytohubba plugin in Cytoscape v3.10.2 was used to identify the top 20 hub genes. Protein-protein interaction networks were then constructed using the STRING database.

### Molecular docking

The SLAMF8 and NINJ2 structures were acquired from the AlphaFold Protein Structure Database. The interaction between SLAMF8 and NINJ2 was investigated through molecular docking using the HDOCK server^17^. Visualization, analysis, and mapping of the best predicted binding mode (lowest score) were carried out using the Pymol program (version 4.3.0).

### Animals

APP/PS1 transgenic mice were acquired from HFK Bioscience Cooperation (14002a, Beijing, China) using the C57BL/6 genetic background. The SLAMF8 knockout (KO) mice were produced by Cyagen (Jiangsu, China). Over 10 generations of backcrossing between SLAMF8 knockout mice and C57BL/6 mice were conducted to guarantee a genetic background of over 99% C57BL/6, which was verified by microsatellite analysis. Backcrossed SLAMF8 knockout (KO) mice were subsequently crossed with APP/PS1 mice to produce the following groups: wild-type (SLAMF8+/+), APP/PS1 (SLAMF8+/+), APP/PS1/SLAMF8 knockout (SLAMF8−/−). All mice were kept in controlled environments at a temperature of 22 °C, with a fixed 12-hour light-dark cycle, and were provided with unrestricted access to food and water. Under permit No. 2024PS149K, the Animal Ethics Committee of China Medical University granted approval for all animal experiments, which were carried out in accordance with the ARRIVE principles. Mice were anesthetized using isoflurane (1–3% for induction and maintenance) for all surgical procedures and sacrificed using carbon dioxide (CO₂) inhalation followed by cervical dislocation to ensure humane treatment. Furthermore, all experimental procedures were performed in strict accordance with relevant guidelines and regulations.

### Behavioral assessment

Spatial memory development was evaluated in age-matched 16-month-old wild-type (SLAMF8+/+), APP/PS1 (SLAMF8+/+), and APP/PS1/SLAMF8 knockout (SLAMF8−/−) mice using the Morris Water Maze test (Morris, 1984). A conventional black circular tank measuring 150 cm in diameter, 70 cm in height, and 45 cm in water depth, filled to a depth of 30 cm with water kept at a temperature of 22 ± 1 °C, constitutes the Morris water maze. The tank is partitioned into four quadrants, including northeast (NE), northwest (NW), southeast (SE), and southwest (SW), utilizing the EthoVision tracking system software (Noldus Information Technology, Wageningen, The Netherlands)^18^. Within the SE quadrant, a concealed platform is positioned at the centre, submerged at a depth of around 2 cm below the water surface. Diverse images function as supplementary visual indicators within the maze and are strategically placed around the tank.

**Fig. 1:**
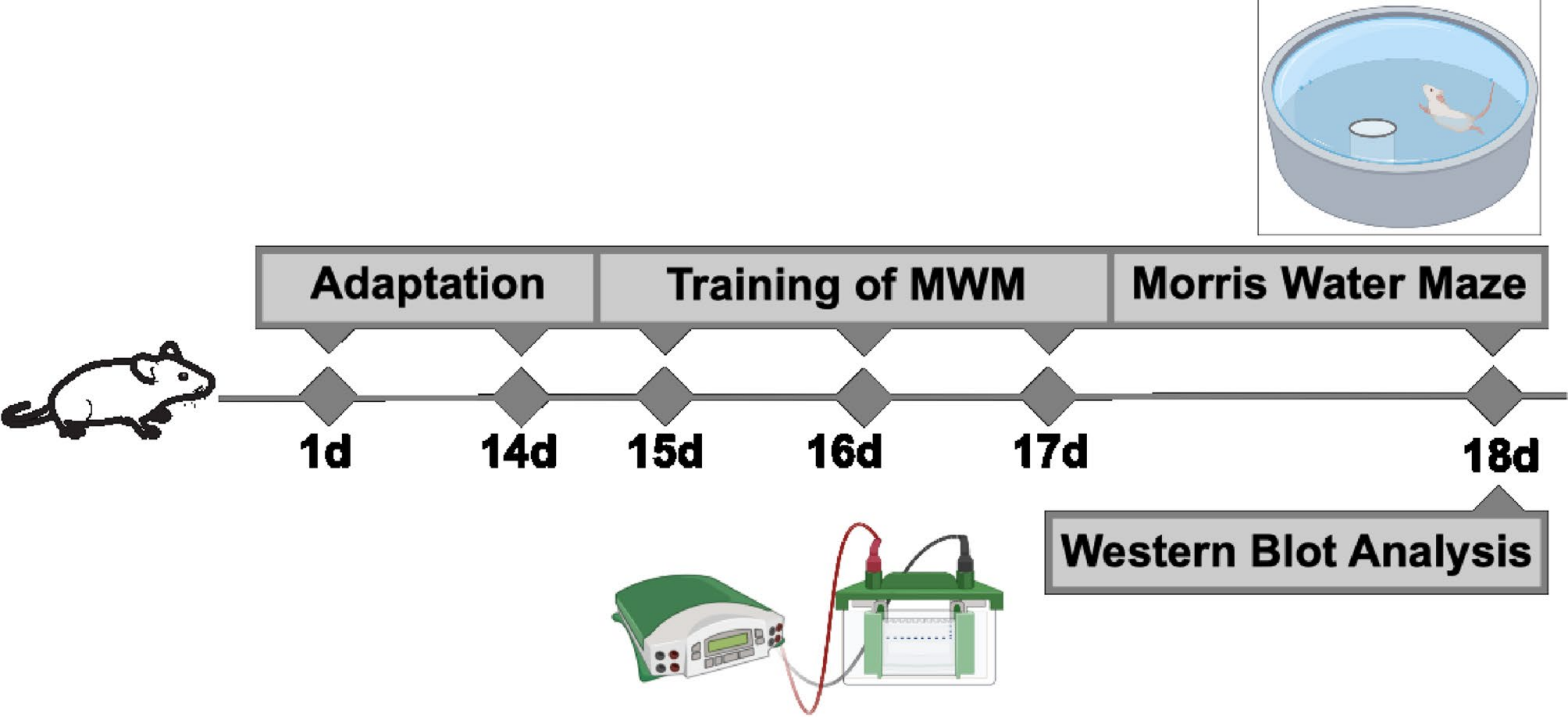
Experimental schedule schematic illustration. The phase of adaptation lasted 2 weeks. Learning and memory capacities were assessed by the Morris water maze tests conducted on days 15 to 18. Following the behavioral studies, brain and hippocampal samples were obtained for subsequent assays (created using Biorender.com).

The examination consists of two stages: location navigation (skill acquisition) and probe. The location navigation phase consists of four repeated trials conducted daily over a span of three consecutive days. During each trial, the animal is immersed in the water, positioned towards the pool wall in one of the quadrants, and given a maximum of 60 s to locate the concealed platform. Failure of an animal to find the platform within the designated time is met with manual guidance and a 20-second period of time spent there to enhance learning. The investigation phase takes place on the fourth day, precisely 24 h following the last learning trial, when the educational platform is no longer available. It is anticipated that each rat will swim for a duration of 60 s, with the greatest amount of time spent in the quadrant where the platform was previously positioned, known as the target quadrant. Behavioral parameters, including distance travelled, escape latency (time to locate the platform), swimming speed, time spent in the target quadrant, and number of platform crossings during the probe phase, are captured by a video camera and analysed using the EthoVision program. After completing the behavioral assessments, the mice were thoroughly sedated and administered 15 ml of phosphate-buffered saline through transcardial perfusion. Subsequently, the brains were removed from the skull. The experimental procedure is illustrated schematically in Fig. 1.

### Materials

The preparation of Aβ_1−42_ oligomers was based on the method established by Klein^19^. The lyophilized synthetic Aβ_1−42_ peptides (107761-42-2, Abcam Co., Ltd., Cambridge, UK) were dissolved in a solution of 1,1,1,3,3,3-hexafluoro-2-propanol (105228, Sigma-Aldrich Corporation, St. Louis, MO, USA), dried by evaporation, and then stored at a temperature of − 80 °C. In preparation for use, the desiccated peptides were dissolved in anhydrous DMSO to a concentration of 5 mM. To prepare Aβ1–42 oligomers, 5 mM Aβ1–42 in DMSO was diluted to 100 µM with ice-cold, phenol red-free Ham’s F-12 medium. The mixture was vortexed for 30 s and incubated at 4 °C for 24 h (Supplementary Fig. 1).

### Cell cultures

The SH-SY5Y cells (CL-0208) were purchased from Procell Life Science & Technology Co., Ltd. (Wuhan, China), and cultivated in a culture medium prepared specifically for SH-SY5Y (CM-0208, Procell). The cells were incubated at 37 °C in a humidified environment with 5% CO2 for 24 h. Next, the cells were exposed to 5 µM Aβ_1−42_ oligomers for a duration of 24 h.

The HMC3 cells (CL-0620) were purchased from Procell (Wuhan, China), and cultivated in a culture medium specifically designed for HMC3 cells (CM-0620, Procell). The cells were incubated at 37 °C in a humidified environment with 5% CO2 for 24 h. Furthermore, the cells were exposed to a concentration of 0.1 µM LPS (L5293, Sigma-Aldrich) for a duration of 24 h.

### RNA isolation and quantitative PCR (qPCR) analyses

Total RNA was extracted from cells using TRIzol™ Reagent (Thermo Fisher Scientific) according to the manufacturer’s protocol. The isolated RNA was quantified and assessed for purity using a spectrophotometer. cDNA was synthesized from 1 µg of RNA using the HiScript^®^ II Q RT SuperMix for qPCR (+ gDNA wiper) (R223-01, VAZYME). Quantitative PCR (qPCR) was performed using ChamQ Universal SYBR qPCR Master Mix (Q711-02, VAZYME) on a 7500HT Fast Real-Time PCR System (Applied Biosystems; Thermo Fisher Scientific). The specific primers utilized for each gene are listed in Table 1. Gene expression levels were normalized to GAPDH as an internal control and quantified using the 2^−ΔΔCt^ method.

### Gene overexpression or knockdown

Cells were transduced with PLV-puro vectors (OriGene) containing the full-length open reading frame (ORF) of SLAMF8. Stable SLAMF8-expressing cell lines were generated after a minimum of two weeks of puromycin selection (58-58-2, Sigma-Aldrich). For gene knockdown, SLAMF8 expression was suppressed using SLAMF8-specific shRNA, while a non-targeting shRNA was used as a control (Generalbiol, Anhui, China). Additionally, siRNA targeting NINJ2 (siNINJ) was introduced into cells using Lipofectamine 2000 (Life Technologies). The specific target sequences are provided in Table 1. After 48 h of transfection or shRNA expression, cells were harvested for downstream analyses, and transfection efficiency was assessed using RT-qPCR and Western blot analysis.

**Table 1:** Primers and ShRNA.

SLAMF8 primers	Forward 5′-AGCCCTACTTCCCATTACAGT-3′ Reverse 5′-AGAGATCGCCAGATAGCCTCA-3′
*NINJ2* primers	Forward 5ʹ-ATGCGGCTGAAGGCGGTGCTG-3ʹ Reverse 5ʹ-TGGCTGCGTTGTTGAGCTGGTTG-3ʹ
*GAPDH* primers	Forward 5ʹ-GGAGCGAGATCCCTCCAAAAT-3ʹ Reverse 5ʹ-GGCTGTTGTCATACTTCTCATGG-3ʹ
shSLAMF8	5ʹ-GAAAGTGACTCTCTGAGAACC-3ʹ
siNIJI2	5ʹ-UUGCACCUGGAGAACCUGAA-3ʹ

### Western blotting

Cell lysates were prepared using RIPA buffer (P0013B, Beyotime, Shanghai, China), followed by centrifugation at 12,000 x *g* for 15 min at 4 °C to clear the debris. The protein content in the supernatants was quantified using a BCA protein assay. Equal protein amounts were then subjected to SDS-PAGE and subsequently transferred onto PVDF membranes. The membranes were blocked with 5% non-fat milk in TBST for 1 h at room temperature, followed by an overnight incubation at 4 °C with the primary antibodies. After washing, HRP-conjugated secondary antibodies were applied for 1 h at room temperature. Detection of protein bands was carried out using a Bio-Rad imaging system (Hercules, CA, USA), and ImageJ software was used for densitometric analysis, with normalization to β-actin levels. The specific primary antibodies used are listed in Table 2.

**Table 2:** Antibodies used in this study.

Antibodies	Source	Identifier
Anti-SLAMF8	Novus	Cat#NBP2-26110
Anti-NINJ2	R&D Systems	Cat#AF5056
Anti-NOX2	Abcam	Cat#ab310337
Anti-NOX4	Abcam	Cat#ab133303
P65	Thermo Fisher Scientific	Cat#51-0500
p-p65	Thermo Fisher Scientific	Cat#MA5-15160
p-IKBα	Cell Signaling Technology	Cat#9246
IKBα	Cell Signaling Technology	Cat#4814
Anti-TLR4	Thermo Fisher Scientific	Cat#MA5-16216
Anti-β-actin	Cell Signaling Technology	Cat#3700

### Enzyme-linked immunosorbent assay (ELISA)

HMC3 cells were treated with 1 µg/mL of lipopolysaccharide (LPS) for 24 h. After incubation, the supernatants were collected and stored at − 80 °C until analysis. The concentrations of IL-1β, IL-6, and TNF-α in the supernatants were measured using commercial ELISA kits specific for IL-1β (EK0394, Boster, Wuhan, China), IL-6 (EK0411, Boster, Wuhan, China), and TNF-α (EK0527, Boster, Wuhan, China), following the manufacturer’s protocols.

### ROS detection

Intracellular reactive oxygen species (ROS) levels were measured using DCFH-DA (D6883, Sigma-Aldrich), while mitochondrial ROS levels were assessed using MitoSOXTM Red reagent (M36008, Thermo Fisher Scientific), following the method described by Kauffman et al. (2016). SH-SY5Y cells treated with 5 µM Aβ_1−42_ were incubated with 10 µM DCFH-DA for 30 min at 37 °C in the dark to detect intracellular ROS. For mitochondrial ROS detection, cells were incubated with 5 µM MitoSOX™ Red reagent under the same conditions. After incubation, cells were washed with PBS and imaged using a confocal laser-scanning microscope (Leica, Wetzlar, Germany). DCFH-DA was excited at 488 nm, and emission was collected at 525 nm. MitoSOX™ Red was excited at 510 nm, with emission collected at 580 nm.

### Co-immunoprecipitation (Co-IP) assay

Cells were lysed in cell lysis buffer (Beyotime, Shanghai, China) on ice for 30 min, followed by centrifugation to clear debris. The supernatant was precleared with rProtein A/G Plus MagPoly beads (RM09008, ABclonal, Wuhan, China) and incubated overnight at 4 °C with antibodies against SLAMF8 or NINJ2. The immune complexes were captured using fresh rProtein A/G Plus MagPoly beads, washed, and eluted for analysis by SDS-PAGE and Western blotting.

### Immunofluorescence assay

SH-SY5Y and HMC3 cells were fixed with 4% paraformaldehyde (MA0192, Meilunbio, Dalian, China) for 20 min at room temperature, followed by permeabilization with 0.1% Triton X-100 (P0096, Beyotime, Shanghai, China) for 10 min. After blocking with 5% BSA in PBS for 1 h, cells were incubated with primary antibodies against SLAMF8 overnight at 4 °C. The next day, cells were thoroughly washed with PBS to remove unbound primary antibodies. They were then incubated with fluorescently labeled secondary antibodies for 1 h at room temperature in the dark to prevent photobleaching. Finally, cells were mounted with antifade medium and imaged using a fluorescence microscope.

### Statistical analysis

Statistical analyses were performed using GraphPad Prism 9.0 (GraphPad Software, CA, USA) and SPSS 19.0 (SPSS Inc., Chicago, USA). Measurements between two groups (mean ± standard deviation) were compared using Student’s t-test. Comparisons among multiple groups were conducted using one-way analysis of variance (ANOVA). Two-way repeated measures ANOVA (Two-way RM-ANOVA) was used to compare different groups across multiple time points. A p-value of < 0.05 was considered statistically significant.

## Results

### SLAMF8 is highly expressed in AD

**Fig. 2:**
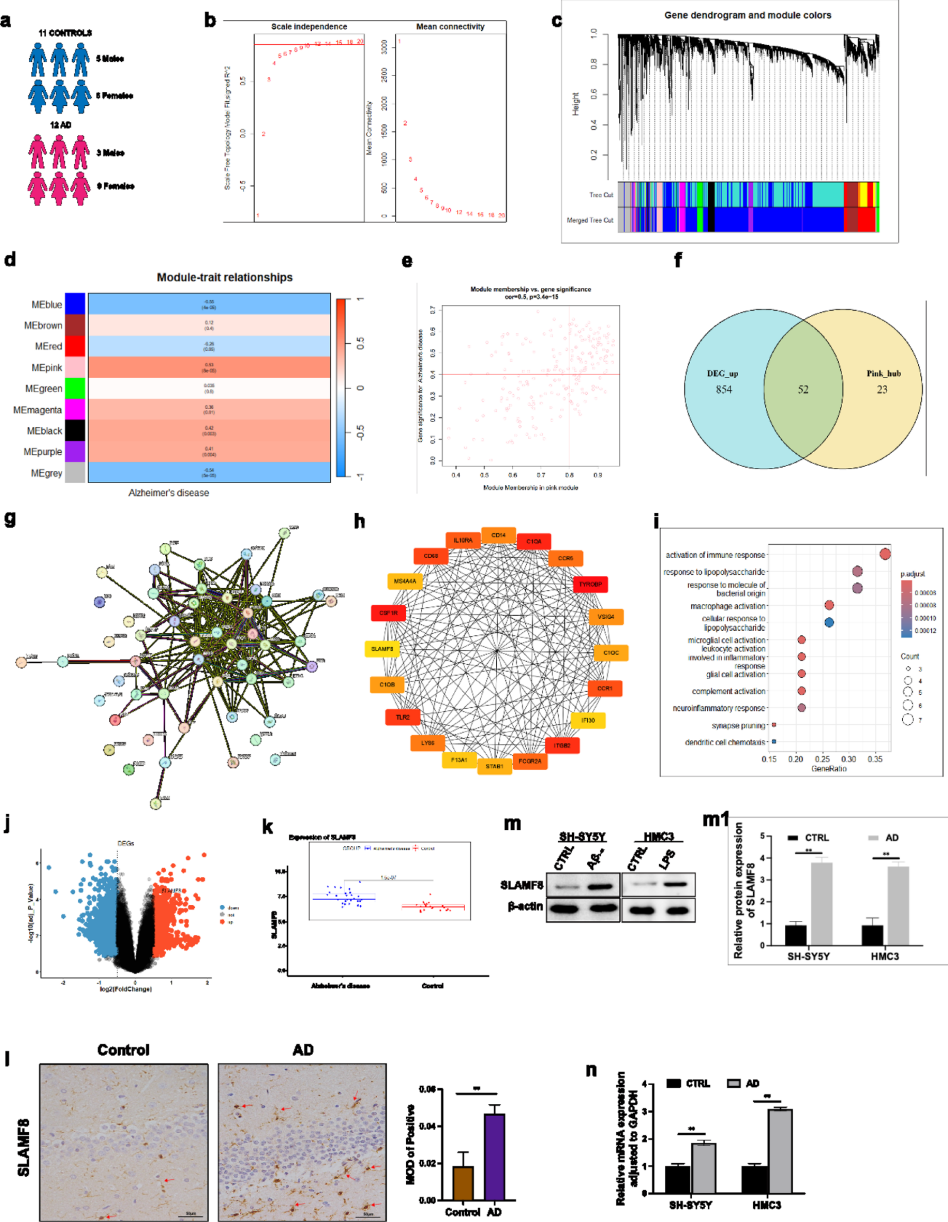
Bioinformatics analysis and experimental validation. (**a**) Screening for differentially expressed genes (DEGs) was conducted in the frontal and temporal cortex samples from 12 Alzheimer’s disease (AD) patients compared to 11 non-demented controls, using data from the GSE122063 dataset. (**b**) Left Panel: The scale-free topology model was evaluated across different soft-threshold powers (β). Right Panel: The mean connectivity was assessed across various soft-threshold powers to determine the optimal β for constructing the network. (**c**) A dendrogram was generated, clustering all genes based on the topological overlap measure (TOM). The branches represent groups of highly interconnected genes, with different colors indicating distinct gene modules. (**d**) The correlation between module eigengenes and AD status was analyzed, identifying significant associations. (**e**) The highlighted genes in the pink module had a gene significance (GS) greater than 0.4 and module membership (MM) greater than 0.8. (**f**) The overlap of hub genes in the pink module with upregulated DEGs was shown in a Venn diagram, identifying 52 key genes. (**g**) The STING database was used to predict the interaction network among these 52 key genes. (**h**) CytoHubba analysis was applied to identify the top 20 genes with the strongest interactions. (**i**) GO enrichment analysis identified that these 20 hub genes are mainly engaged in immune and inflammatory response pathways. (**j**) A volcano plot indicated that SLAMF8 is significantly upregulated in AD patients. (**k**) A box plot further confirmed the significant upregulation of SLAMF8 in AD patients. (**l**) SLAMF8 protein was found to be significantly upregulated in the hippocampal tissue of APP/PS1 mice relative to WT mice, as shown by immunohistochemistry (IHC) staining. The increased expression of SLAMF8 in Aβ_1−42_-treated SH-SY5Y cells and LPS-treated HMC3 cells was confirmed by both Western blot (**m**) and RT-PCR (**n**).

Transcriptome data from GSE122063, including frontal and temporal cortex tissue samples from 12 Alzheimer’s disease (AD) patients (3 males and 9 females) and 11 non-demented controls (5 males and 6 females) (Fig. 2a), were utilized for further analysis. Gene co-expression networks were constructed using the WGCNA package. (Fig. 2b), through which multiple co-expression modules were identified via gene clustering (Fig. 2c). Correlation analysis between gene modules and AD status revealed that the pink module (MEpink, *p* < 0.001, *r* = 0.53) was significantly positively correlated with AD (Fig. 2d). Using gene module membership (GM > 0.8) and gene significance (GS > 0.4) as criteria, we identified key genes within the pink module (Fig. 2e). From these genes, 52 upregulated differentially expressed genes were identified (Fig. 2f), and their interactions were predicted using the STING database (Fig. 2g). Further analysis using cytoHubba extracted the top 20 genes with the strongest interactions (Fig. 2h). GO enrichment analysis of these 20 genes revealed their predominant involvement in immune and inflammatory response pathways (Fig. 2i).

Additionally, differential expression analysis demonstrated that SLAMF8 is significantly upregulated in AD groups (Fig. 2j, k). To further explore the differential expression of SLAMF8, classic preclinical AD models were utilized, including Aβ_1−42_-treated SH-SY5Y cells^20^ and LPS-treated HMC3 microglia^21^. SLAMF8 was found to be highly expressed at both the protein (Fig. 2m,m1) and mRNA levels (Fig. 2n) in these models. This finding was further supported by immunohistochemistry (IHC) staining, which demonstrated a significant increase in SLAMF8 protein expression in the hippocampus of APP/PS1 mice compared to WT mice (Fig. 2l).

### Ectopic expression of SLAMF8 promoted neuroinflammation and oxidative stress

**Fig. 3:**
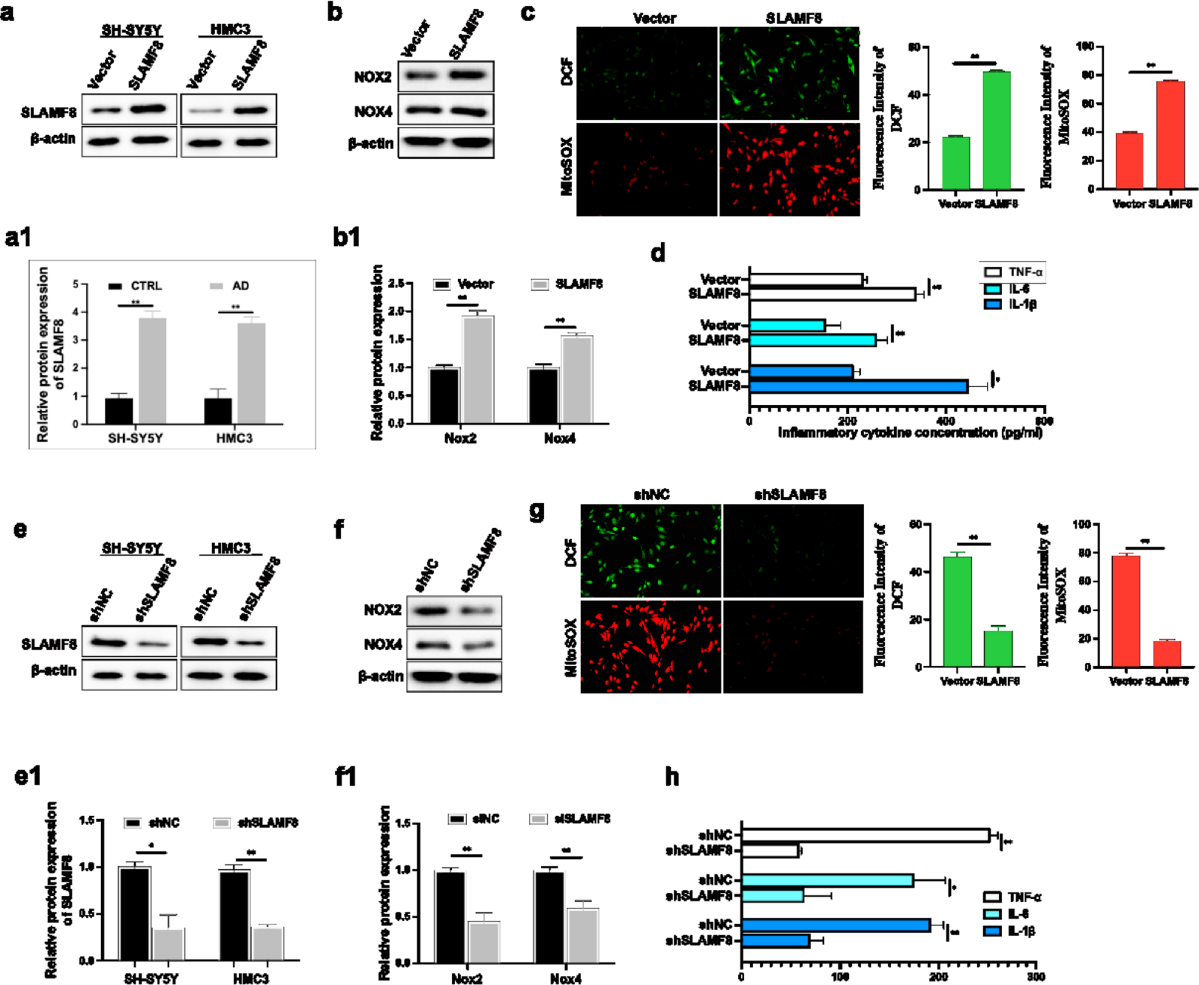
(**a**) Western blot confirmed the overexpression of SLAMF8 in Aβ_1−42_-treated SH-SY5Y cells and LPS-treated HMC3 cells. (**a1**) Quantification of Western blot results was performed and normalized against β-actin (*n* = 3). (**b**) Overexpression of SLAMF8 increased Nox2 and Nox4 expression in Aβ_1−42_-treated SH-SY5Y cells. (**b1**) Quantification of Western blot results was performed and normalized against β-actin (*n* = 3). (**c**) SLAMF8 expression significantly increased cellular ROS production and mitochondrial ROS levels in Aβ_1−42_-treated SH-SY5Y cells, as demonstrated by the fluorescence signals of DCF and MitoSOX Red. (**d**) SLAMF8 expression significantly increased neuroinflammation in LPS-treated HMC3 cells (*n* = 3). (**e**) Knockdown of SLAMF8 by sh-SLAMF8 in Aβ_1−42_-treated SH-SY5Y and LPS-treated HMC3 cells was confirmed by western blot. (**e1**) Quantification of Western blot results was performed and normalized against β-actin (*n* = 3). (**f**) Knockdown of SLAMF8 reduced Nox2 and Nox4 expression in Aβ_1−42_-treated SH-SY5Y cells. (**f1**) Quantification of Western blot results was performed and normalized against β-actin (*n* = 3). (**g**) SLAMF8 knockdown significantly decreased cellular ROS production and mitochondrial ROS levels in Aβ_1−42_-treated SH-SY5Y cells. (**h**) SLAMF8 knockdown decreased the levels of inflammation-related proteins, including IL-1β, IL-6, and TNF-α (*n* = 3). Data obtained from measurements are reported as mean ± standard deviation. Multiple groups were compared using one-way analysis of variance (ANOVA) with Tukey’s post hoc test, and comparisons between two groups were conducted using a paired t-test. The symbol “*” denotes *p* ≤ 0.05, while “**” denotes *p* ≤ 0.01.

To explore the role of SLAMF8 in AD, we stably introduced SLAMF8 expression vectors or control vectors into AD cell models (Aβ_1−42_-treated SH-SY5Y cells and LPS-treated HMC3 cells). Western blot and quantitative analysis confirmed that the protein levels of SLAMF8 were elevated in the SLAMF8 overexpression group compared to the control group. (Fig. 3a, a1). Overexpression of SLAMF8 significantly increased mitochondrial superoxide production and oxidative stress in Aβ_1−42_-treated SH-SY5Y cells (Fig. 3b, b1, c) and promoted neuroinflammation in LPS-treated HMC3 cells (Fig. 3d). Conversely, knockdown of SLAMF8 in both Aβ_1−42_-treated SH-SY5Y cells and LPS-treated HMC3 cells (Fig. 3e,e1) significantly inhibited mitochondrial superoxide production and oxidative stress in Aβ_1−42_-treated SH-SY5Y cells (Fig. 3f, f1, g) and decreased neuroinflammation in LPS-treated HMC3 cells (Fig. 3h).

### SLAMF8 activated TLR4/NF-κB signaling

**Fig. 4:**
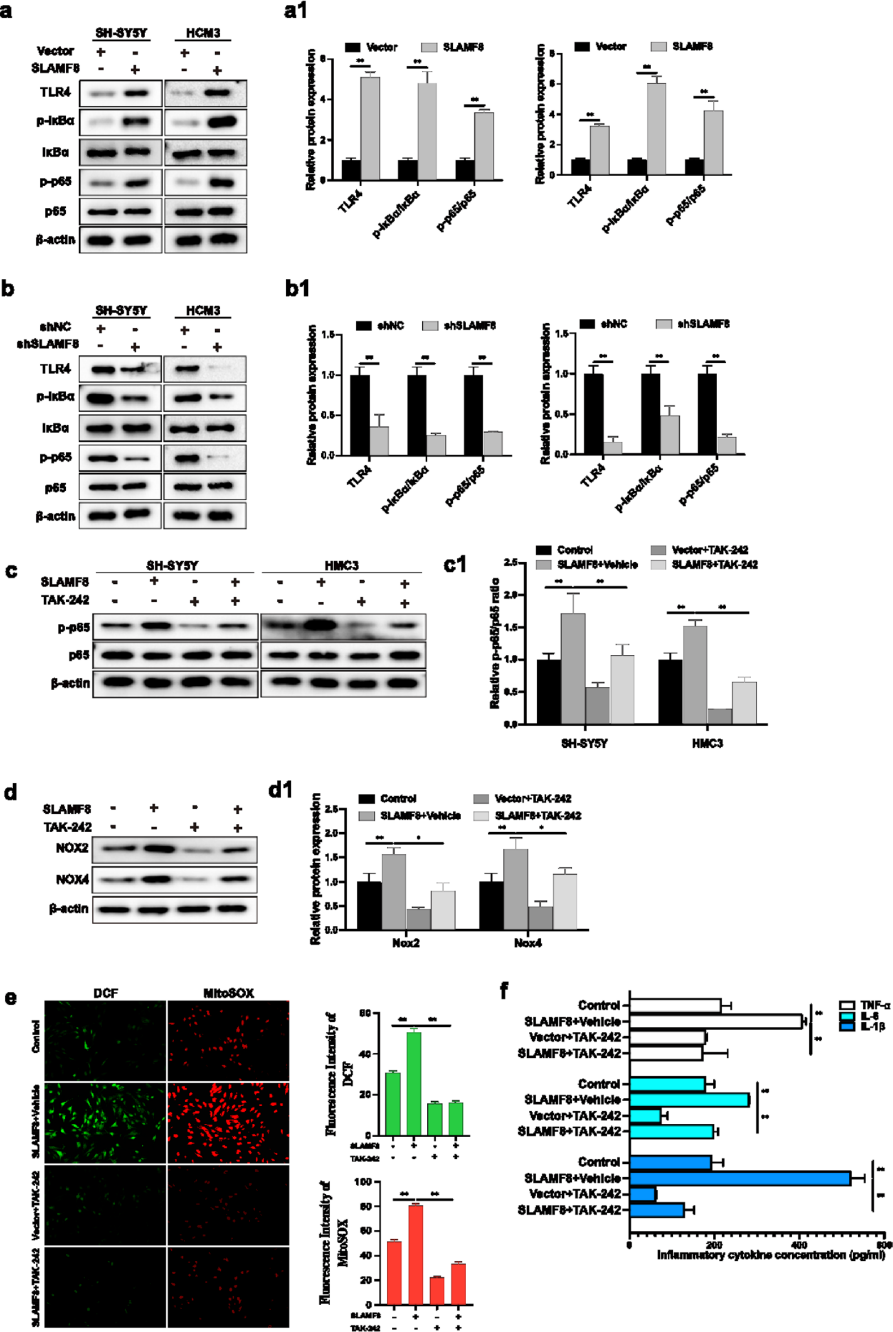
(**a**) The overexpression of SLAMF8 in AD cell models increased the levels of TLR4/NF-κB signaling-related markers (TLR4, p-IκBα/IκBα ratio) and the phospho-p65/p65 ratio, as confirmed by Western blot. Quantification of the Western blot results was performed and normalized against β-actin (*n* = 3) (**a1**). (**b**) Conversely, knockdown of SLAMF8 produced the opposite effect and the data were quantified and normalized to β-actin (*n* = 3). (**b1**) The TLR4/NF-κB signaling inhibitor TAK-242 suppressed TLR4 expression (**c**,**c1**) and mitigated the increase in ROS (**d**,**e**) and neuroinflammation (**f**) caused by SLAMF8 overexpression in AD cell models. Data are presented as mean ± standard deviation. One-way ANOVA with Tukey’s post hoc test was used for multiple group comparisons, and a paired t-test was used for comparisons between two groups. “*” indicates *p* ≤ 0.05, and “**” indicates *p* ≤ 0.01.

To gain deeper insights into the regulatory mechanisms of SLAMF8 in Alzheimer’s disease (AD), we assessed the expression levels of Toll-like receptor 4 (TLR4), phosphorylated p65 (phospho-p65), total p65, phosphorylated IκBα (phospho-IκBα), and total IκBα across different experimental groups. The findings revealed that SLAMF8 activated the TLR4/NF-κB signaling pathway (Fig. 4a, a1). Overexpressing SLAMF8 led to an upregulation of TLR4, an increased p-IκBα/IκBα ratio, and a higher phospho-p65/p65 ratio. Conversely, silencing SLAMF8 reversed these effects (Fig. 4b, b1). To assess whether SLAMF8’s role in AD is mediated through TLR4/NF-κB activation, we treated Aβ_1−42_-exposed SH-SY5Y cells and LPS-stimulated HMC3 cells, with and without SLAMF8 overexpression, with TAK-242, a selective TLR4 inhibitor that blocks NF-κB activation. TAK-242 effectively reduced TLR4 expression (Fig. 4c, c1) and mitigated the pro-inflammatory and oxidative stress effects induced by SLAMF8, as indicated by reduced ROS levels (Fig. 4d, d1, e) and lower neuroinflammatory markers (Fig. 4f).

### Interaction between SLAMF8 and NINJ2

**Fig. 5:**
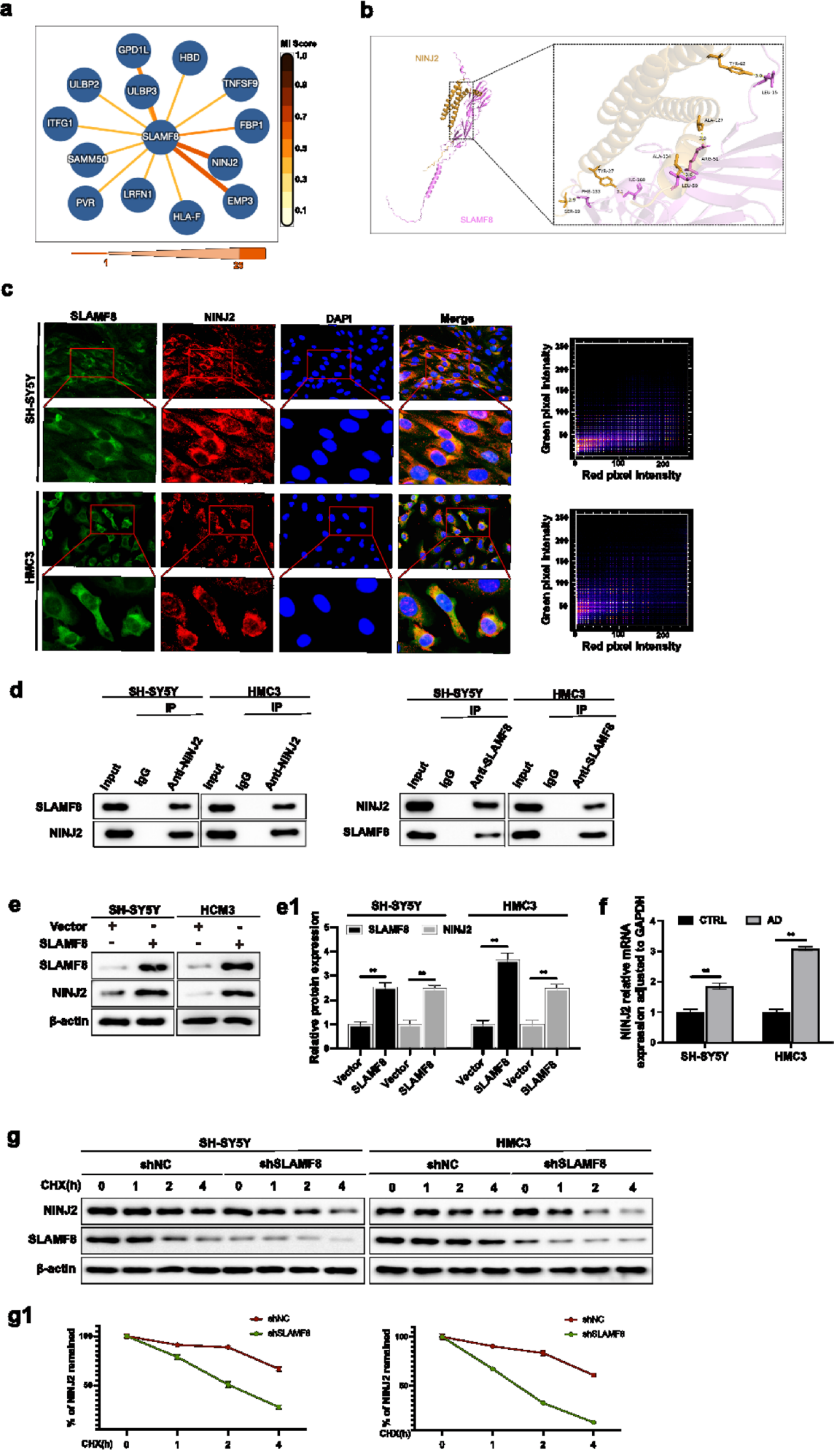
(**a**) The IntAct database (https://www.ebi.ac.uk/intact/home) displays the interaction network of SLAMF8 with multiple proteins. (**b**) The binding mode of SLAMF8 and NINJ2 was predicted using HDOCK. Left: cartoon view of NINJ2 (orange) bound to SLAMF8 (purple). Right: detailed interaction network showing key residues of NINJ2 and SLAMF8 as sticks. H-bonds are indicated by yellow dashed lines, with labeled distances. (**c**) Immunofluorescence assays were used to examine the localization of SLAMF8 and NINJ2 in cells (upper panel: scale bar = 20 μm; lower panel: scale bar = 5 μm). (**d**) Western blot analysis following Co-immunoprecipitation (Co-IP) verified the interaction between SLAMF8 and NINJ2 in both Aβ_1−42_-treated SH-SY5Y and LPS-treated HMC3 cells. (**e**) SLAMF8 overexpression elevated NINJ2 protein levels in AD cell models, with results quantified and normalized to β-actin (*n* = 3) (**e1**). (**f**) The mRNA level of NINJ2 was not altered by the overexpression of SLAMF8 in Aβ_1−42_-treated SH-SY5Y and LPS-treated HMC3 cells. (**g**) SLAMF8 increased the stability of NINJ2 in Aβ_1−42_-treated SH-SY5Y and LPS-treated HMC3 cells. (**g1**) The percentage of NINJ2 that remained was calculated. Data are presented as mean ± standard deviation. One-way ANOVA with Tukey’s post hoc test was used for multiple group comparisons, and a paired t-test was used for comparisons between two groups. “*” indicates *p* ≤ 0.05, and “**” indicates *p* ≤ 0.01.

To further determine the downstream targets of SLAMF8, we queried the IntAct database (https://www.ebi.ac.uk/intact/home)^22^, which displays the interaction network of SLAMF8 with multiple proteins proteins (Fig. 5a). Among the potential interacting proteins, NINJ2 is a membrane protein involved in nerve regeneration and has been implicated in various neuroinflammatory processes. It plays a role in cellular adhesion and may influence signaling pathways related to inflammation and neurodegeneration. Additionally, NINJ2 was reported to potentially interact with SLAMF8, making it the most interesting candidate for interaction with SLAMF8^23^.

Molecular docking analysis indicated a possible interaction between SLAMF8 and NINJ2 (Fig. 5b). Immunofluorescence analysis showed that SLAMF8 and NINJ2 were partially colocalized in SH-SY5Y cells, HMC3 cells (Fig. 5c), and the APP/PS1 mouse hippocampus (Supplementary Fig. 2). To confirm the interaction between SLAMF8 and NINJ2, co-immunoprecipitation (Co-IP) experiments were conducted on Aβ_1−42_-treated SH-SY5Y cells and LPS-treated HMC3 cells. More NINJ2 was detected by the anti-SLAMF8 antibody compared to the IgG control, and more SLAMF8 was detected by the anti-NINJ2 antibody (Fig. 5d), indicating a direct interaction between SLAMF8 and NINJ2. Interestingly, although SLAMF8 overexpression upregulated NINJ2 protein levels (Fig. 5e, e1), it did not affect NINJ2 mRNA levels (Fig. 5f). Given this, we next investigated whether SLAMF8 influences the stability of NINJ2. Cells transfected with shSLAMF8 or control shNC were treated with cycloheximide, a protein synthesis inhibitor. The results indicated that the presence of SLAMF8 in these cells enhanced NINJ2 stability (Fig. 5g, g1).

### NINJ2’s pathogenic function via interaction with SLAMF8

**Fig. 6:**
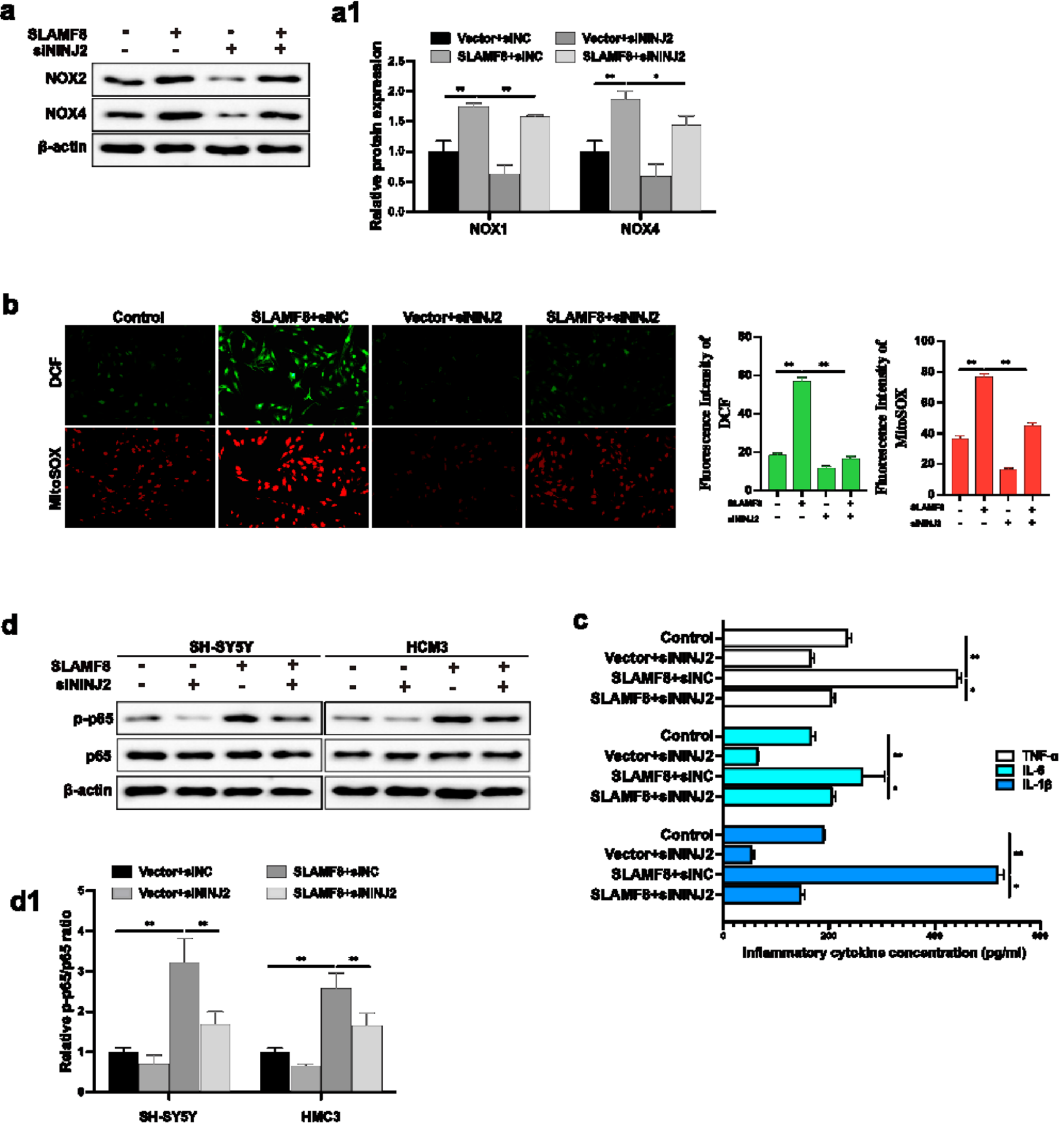
(**a**,**b**) Knockdown of NINJ2 significantly abolished SLAMF8-induced oxidative stress, reduced ROS levels (DCF and MitoSOX Red), and decreased NOX2 and NOX4 expression in Aβ1-42-treated SH-SY5Y cells. (**c**) Similarly, knockdown of NINJ2 significantly reduced the SLAMF8-treated neuroinflammation in LPS-treated HMC3 cells. (**d**) In SLAMF8-stably overexpressing AD cell models, transient knockdown of NINJ2 with siNINJ2 for 48 h significantly abolished SLAMF8-induced p65 phosphorylation in the TLR4/NF-κB signaling pathway. Data are presented as mean ± standard deviation. One-way ANOVA with Tukey’s post hoc test was used for multiple group comparisons, and a paired t-test was used for comparisons between two groups. “*” indicates *p* ≤ 0.05, and “**” indicates *p* ≤ 0.01..

To explore the impact of NINJ2 on SLAMF8-mediated neuroinflammation and oxidative stress, we transfected AD cell models with SLAMF8 or control vectors, followed by co-transfection with siRNA targeting NINJ2. The knockdown of NINJ2 nullified the enhancing effect of SLAMF8 on ROS production in Aβ_1−42_-treated SH-SY5Y cells (Fig. 6a, a1,b), as well as the neuroinflammatory response in LPS-treated HMC3 cells (Fig. 6c). We next investigated whether SLAMF8 activates the TLR4/NF-κB signaling pathway via NINJ2 mediation. Consistent with our expectations, knockdown of NINJ2 suppressed p65 phosphorylation in these cells (Fig. 6d, d1). Taken together, these findings indicate that SLAMF8’s pathogenic role and its activation of the TLR4/NF-κB signaling pathway are at least partly reliant on NINJ2 expression in AD cell models.

### SLAMF8 gene deficiency protects against spatial learning and memory impairments in APP/PS1 mice

**Fig. 7:**
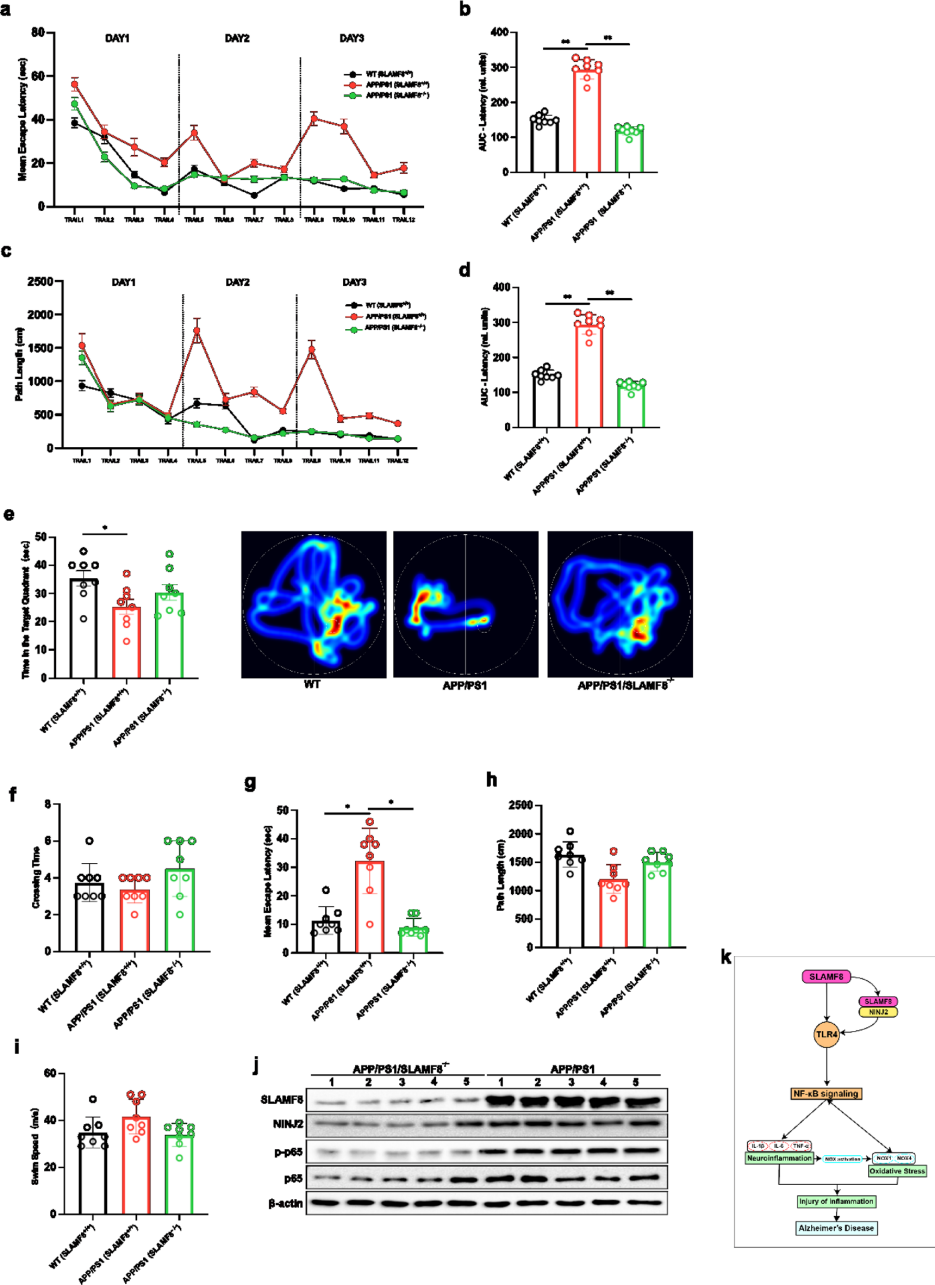
Results from the MWM (Morris water maze) demonstrate that SLAMF8 gene deficiency attenuates the progression of spatial learning and memory deficits in APP/PS1 mice. During the learning phases, (**a**) escape latency (sec) and its corresponding (**b**) area-under-the-curve (AUC), and (**c**) distance travelled (cm) and its corresponding (**d**) area-under-the-curve (AUC) were measured for WT (SLAMF8+/+), APP/PS1 (SLAMF8+/+), and APP/PS1/SLAMF8 KO (SLAMF8−/−) mice (mean ± SEM; for (**a**,**c**), two-way RM-ANOVA; for (**b**,**d**), one-way ANOVA, *p < 0.05, **p < 0.01). During the probe phase, (**e**) time spent in the target quadrant, (**f**) number of crossings over the previously hidden platform, (**g**) escape latency, (**h**) overall swimming distance, and (**i**) overall swimming speed were measured for all groups of mice (mean ± SEM, *p < 0.05, **p < 0.01). (**j**) Western blot analysis confirmed that SLAMF8 gene deficiency in APP/PS1 mice decreased the expression of NINJ2 and phospho-p65, with levels quantified and normalized to β-actin (**j1**). (**k**) A schematic illustration of the molecular mechanism of SLAMF8-NINJ2 interaction in the TLR4/NF-κB pathway in Alzheimer’s disease is provided.”

To investigate the effect of SLAMF8 on learning tasks and hippocampus-dependent memory, we utilized the Morris Water Maze (MWM) to assess spatial memory in mice after a 14-day adaptation period. During the 3-day cue training, all experimental groups demonstrated a progressive decrease in both escape latency and swimming distance (Fig. 7a, c). However, the APP/PS1 (SLAMF8+/+) group showed a slower and less pronounced reduction in these metrics compared to the other groups. Notably, the APP/PS1 (SLAMF8+/+) mice exhibited significantly longer escape latency compared to control mice, while APP/PS1 (SLAMF8−/−) mice displayed a significant reduction in escape latency (Fig. 7b). Additionally, the learning curve, analyzed by path length, revealed significant effects of both time (indicating learning performance over days) and group differences, as determined by repeated-measures ANOVA (Fig. 7c).

AUC analysis demonstrated that APP/PS1 (SLAMF8+/+) mice traveled significantly longer distances compared to control mice during the 3-day acquisition phase, whereas APP/PS1 mice with SLAMF8 gene deficiency (SLAMF8−/−) significantly reduced this increase in path length (Fig. 7d). In the probe trial, APP/PS1 (SLAMF8+/+) mice spent significantly less time in the target quadrant searching for the platform compared to controls, while APP/PS1 mice with SLAMF8 gene deficiency exhibited an increased duration in this quadrant (Fig. 7e). Additionally, during the probe phase, no significant differences were observed in the number of crossings over the area where the platform was previously hidden among the three groups (Fig. 7f). During the probe phase, APP/PS1 (SLAMF8+/+) mice exhibited a considerable delay in escape latency compared to other groups (Fig. 7g). Despite this delay, one-way ANOVA indicated that there were no significant variations in total swimming distance (Fig. 7h) or swimming speed (Fig. 7i) among the groups on the fourth day of the MWM test.

Furthermore, Western blot analysis of mice brain tissue revealed that both NINJ2 expression and p65 phosphorylation were significantly reduced in the APP/PS1 (SLAMF8−/−) group compared to the APP/PS1 group (Fig. 7j), supporting the mechanisms observed in vitro.

## Discussion

In this study, analysis of the GSE122063 dataset from the Gene Expression Omnibus (GEO) database revealed that SLAMF8 is highly associated with Alzheimer’s disease (AD) and significantly upregulated in AD samples. This differential expression was validated both in vivo and in vitro. RT-PCR and Western blot confirmed elevated SLAMF8 levels in Aβ_1−42_-treated SH-SY5Y cells, LPS-treated HMC3 cells, and APP/PS1 mice. Additionally, immunohistochemistry staining demonstrated significantly higher SLAMF8 protein expression in the hippocampus of APP/PS1 mice.

SLAMF8 is a member of the signaling lymphocyte activation molecule (SLAM) family, which includes nine structurally related proteins. The SLAM family is divided into classical and non-classical categories, with SLAMF1-7 classified as classical members and SLAMF8 and SLAMF9 as non-classical members. Unlike the classical SLAM receptors, the functions of the non-classical SLAM receptors are less well characterized. Limited studies have suggested that SLAMF8 may influence inflammation and oxidative stress^24,25^. In this study, we demonstrated that SLAMF8 expression is significantly upregulated in AD models. Elevated SLAMF8 expression was associated with increased neuroinflammation and oxidative stress, which are both linked to the pathogenesis of AD and may exacerbate disease progression. These results highlight the potential role of SLAMF8 in the progression of Alzheimer’s disease by contributing to neuroinflammation and oxidative stress. Given its upregulation in AD models, SLAMF8 may serve as a valuable biomarker for predicting disease severity and could be a promising target for therapeutic intervention.

Next, we investigated the molecular mechanisms through which SLAMF8 contributes to Alzheimer’s disease (AD) pathology. Emerging evidence has linked SLAMF8 to the TLR4/NF-κB pathway^7^. Our findings further identified this pathway as a critical downstream mediator of SLAMF8’s effects in AD. Overexpression of SLAMF8 activated the TLR4/NF-κB signaling cascade, resulting in elevated TLR4 levels and increased phosphorylation of p65 and IκBα. This activation subsequently upregulated TLR4/NF-κB target genes, amplifying the inflammatory response. The role of the TLR4/NF-κB pathway in AD is well-established, with its activation known to drive neuroinflammation and contribute to disease progression through increased pro-inflammatory cytokine production and oxidative stress^26–28^.

Increasing evidence suggests that proteins rely on multi-protein complexes to exert their functions. Here, we queried the IntAct database and identified NINJ2 as a potential Interacting partner of SLAMF8 in the TLR4/NF-κB signaling pathway. NINJ2 is a transmembrane protein encoded by a gene located on chromosome 12p13. It plays a crucial role in mediating cell-cell interactions during nervous system development and regeneration, particularly by promoting neurite outgrowth and supporting the regeneration of sensory and enteric neurons^11^. Research has shown that NINJ2 is upregulated following nerve injury, where it contributes to neuroinflammation by regulating the NF-κB pathway through its interaction with TLR4^16,29^. In this study, we demonstrated a direct interaction between SLAMF8 and NINJ2 through Co-IP and confocal immunofluorescence. Additionally, we found that overexpression of SLAMF8 led to a significant upregulation of NINJ2, suggesting that NINJ2 may serve as an interacting partner of SLAMF8. Furthermore, knockout of NINJ2 eliminated SLAMF8-mediated TLR4/NF-κB signaling, along with the associated increases in neuroinflammation and oxidative stress. Therefore, the SLAMF8-NINJ2-TLR4/NF-κB axis may represent a newly identified component of the pathogenic mechanism underlying Alzheimer’s disease (Fig. 7k).

In conclusion, our study demonstrates that SLAMF8 and NINJ2 are key drivers of neuroinflammation and oxidative stress in Alzheimer’s disease by activating the TLR4/NF-κB pathway. These findings open new avenues for Alzheimer’s disease research, suggesting that SLAMF8 and NINJ2 could serve as potential therapeutic targets. Further exploration of this may lead to the development of novel treatment strategies for AD.

## Electronic supplementary material

Below is the link to the electronic supplementary material.

Supplementary Material 1

Supplementary Material 2

Supplementary Material 3

## Data Availability

The raw data supporting the conclusions of this article will be made available by the corresponding author upon reasonable request.

## Abbreviations

AD: Alzheimer’s disease
SLAMF8: Signaling lymphocytic activation molecule family member 8
NINJ2: Nerve injury-induced protein 2
TLR4: Toll-like receptor 4
NF-κB: Nuclear factor kappa-light-chain-enhancer of activated B cells
GEO: Gene expression omnibus
RT-PCR: Reverse transcription polymerase chain reaction
APP/PS1: Amyloid precursor protein/presenilin 1 (transgenic mouse model)
LPS: Lipopolysaccharide
Co-IP: Co-immunoprecipitation
IHC: Immunohistochemistry
Aβ_1−42_: Amyloid beta 1–42
ROS: Reactive oxygen species
