# Supplementary material for: SLAMF8 and NINJ2 promote neuroinflammation and oxidative stress through TLR4 NF kappa B pathway in Alzheimer’s disease
Source: Sci Rep. 2025 May 20;15:17501. doi: 10.1038/s41598-025-02097-6 (PMC12092773; doi:10.1038/s41598-025-02097-6)
Supplement: Supplementary file 1 — Supplementary Material 1 [file 41598_2025_2097_MOESM1_ESM.docx]

Figure 2

m.（lift: SH-SY5Y; right: HMC3）


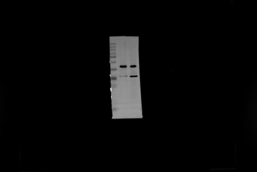

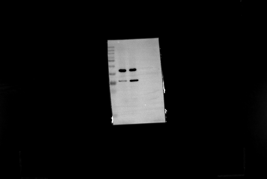

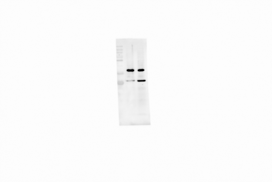

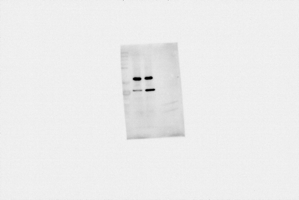


Figure 3

a.（lift: SH-SY5Y; right: HMC3）


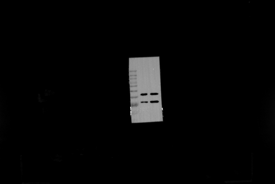

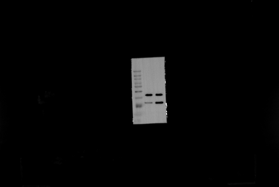

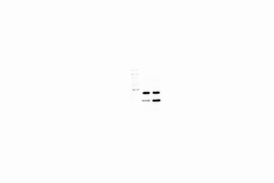

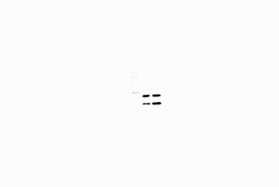


b、f （NOX2，NOX4，β-actin）


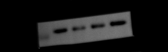

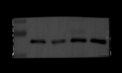

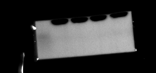

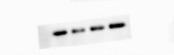

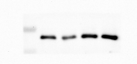

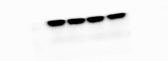


e


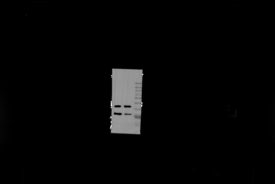

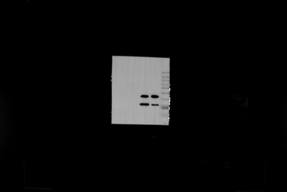

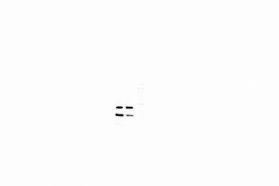

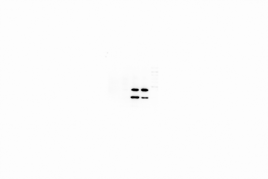


Figure 4

a. (TLR4, p-IκBα, IκBα,p- p65, p65,β-actin)

(SH-SY5Y)


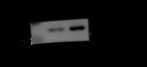

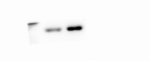

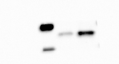

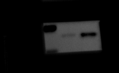

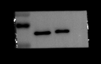

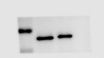

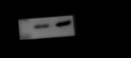

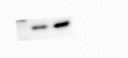

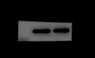

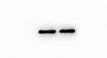

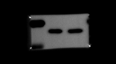

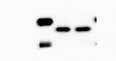


(HMC)


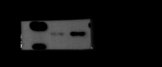

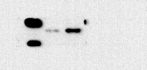

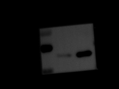

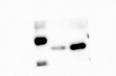

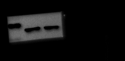

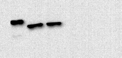

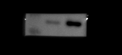

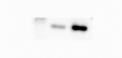

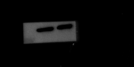

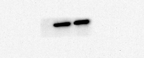

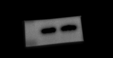

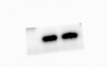


b. (TLR4, p-IκBα, IκBα,p- p65, p65,β-actin)

(SH-SY5Y)


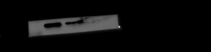

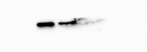

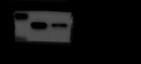

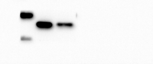

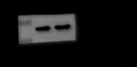

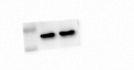

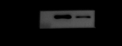

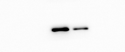

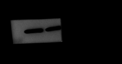

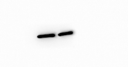

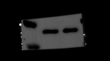

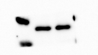


(HMC)


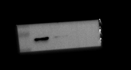

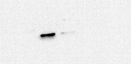

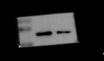

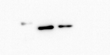

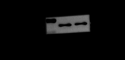

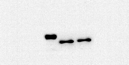

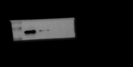

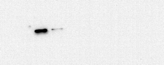

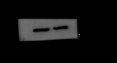

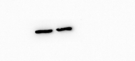

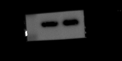

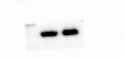


c. (TLR4,β-actin)

(SH-SY5Y)


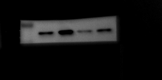

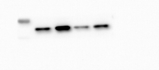

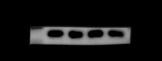

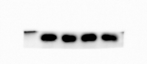


(HMC)


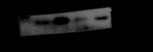

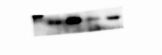

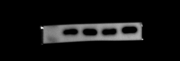

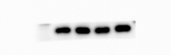


d. (NOX2，NOX4，β-actin)


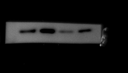

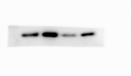

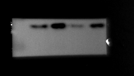

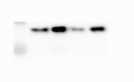

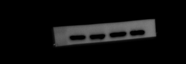

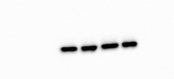


Figure 5

d. (SLAMF8，NINJ2)

(SH-SY5Y)


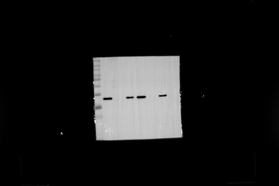

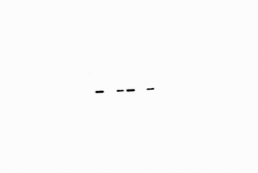

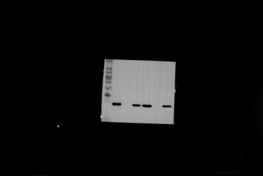

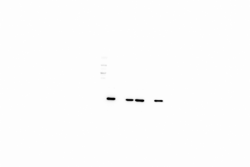


(HMC)


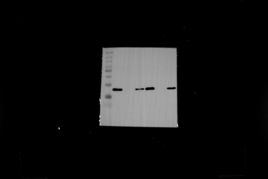

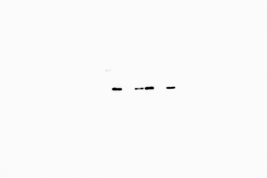

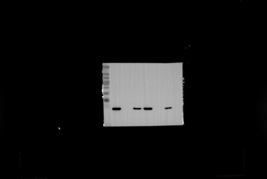

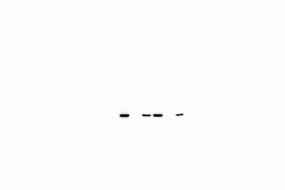


e.

(SH-SY5Y)


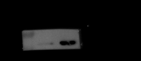

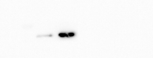

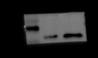

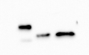

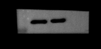

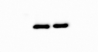


(HMC)


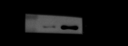

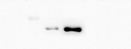

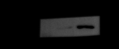

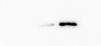

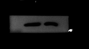

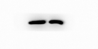


g. (NINJ2, SLAMF8)

(SH-SY5Y)

(HMC)

Figure 6

a. (NOX2，NOX4，β-actin)

d. (p-p65，p65，β-actin)

(SH-SY5Y)

(HMC)

Figure 7

j. (SLAMF8，NINJ2，p-p65，p65，β-actin)
