# Supplementary material for: SLAMF8 and NINJ2 promote neuroinflammation and oxidative stress through TLR4 NF kappa B pathway in Alzheimer’s disease
Source: Sci Rep. 2025 May 20;15:17501. doi: 10.1038/s41598-025-02097-6 (PMC12092773; doi:10.1038/s41598-025-02097-6)
Supplement: Supplementary file 2 — Supplementary Material 2 [file 41598_2025_2097_MOESM2_ESM.docx]

**Supplementary 1**

The preparation of Aβ_1-42_ oligomers was based on the method established by Klein^[19]^. The lyophilized synthetic Aβ_1-42_ peptides (107761-42-2, Abcam Co., Ltd., Cambridge, UK) were dissolved in a solution of 1,1,1,3,3,3-hexafluoro-2-propanol (105228, Sigma-Aldrich Corporation, St. Louis, MO, USA), dried by evaporation, and then stored at a temperature of -80°C. In preparation for use, the desiccated peptides were dissolved in anhydrous DMSO to a concentration of 5 mM.

To prepare Aβ_1–42_ oligomers, 5 mM Aβ_1–42_ in DMSO was diluted to 100 μM with ice-cold, phenol red-free Ham’s F-12 medium (BioSource). The mixture was vortexed for 30 seconds and incubated at 4 °C for 24 h.

The molecular weight was determined by SDS-PAGE. The monomer has a molecular weight of approximately 4.6 kDa; oligomers range from 2 to 8 times the monomer size.

**Figure**

**Western analysis of SDS-PAGE. Representative Western blots of Aβ_1–42_ oligomers incubated for 12 and 24 h. The representative figure shows 12- and 24-h oligomers (lanes 1 and 2).**
