# Supplementary material for: SLAMF8 and NINJ2 promote neuroinflammation and oxidative stress through TLR4 NF kappa B pathway in Alzheimer’s disease
Source: Sci Rep. 2025 May 20;15:17501. doi: 10.1038/s41598-025-02097-6 (PMC12092773; doi:10.1038/s41598-025-02097-6)
Supplement: Supplementary file 3 — Supplementary Material 3 [file 41598_2025_2097_MOESM3_ESM.docx]

**Supplementary 2**

**Figure**

**Immunofluorescence assays were used to examine the localization of SLAMF8 and NINJ2 in mouse hippocampus (upper panel: scale bar = 20 µm; lower panel: scale bar = 5 µm).**
